# Comparative Genome Analysis of Canine *Frederiksenia canicola* Isolates

**DOI:** 10.3390/antibiotics13121235

**Published:** 2024-12-22

**Authors:** Marianna Domán, Krisztina Pintér, Boglárka Dóra Pollák, Ágnes Pintér, Enikő Wehmann, Miklós Tenk, Tibor Magyar

**Affiliations:** 1 HUN-REN Veterinary Medical Research Institute, 1143 Budapest, Hungary; pinter.krisztina@vmri.hun-ren.hu (K.P.); pollak.boglarka@nngyk.gov.hu (B.D.P.); wehmann.eniko@vmri.hun-ren.hu (E.W.); magyar.tibor@vmri.hun-ren.hu (T.M.); 2 Department of Microbiology and Infectious Diseases, University of Veterinary Medicine, 1143 Budapest, Hungary; pinter.agnes000@gmail.com (Á.P.); tenk.miklos@univet.hu (M.T.); 3 National Laboratory of Infectious Animal Diseases, Antimicrobial Resistance, Veterinary Public Health and Food Chain Safety, University of Veterinary Medicine, 1078 Budapest, Hungary

**Keywords:** antimicrobial susceptibility, *Frederiksenia canicola*, genome analysis, resistance, whole-genome sequencing

## Abstract

**Background/Objectives**: The One Health approach is crucial for managing and controlling the spread of antimicrobial resistance. *Frederiksenia canicola* is a recently identified bacterial species that seems to be a component of the oral microbiota of dogs; however, its pathogenic nature is questionable. **Methods**: In this study, the antibacterial susceptibility of *F. canicola* isolates was determined using the disk diffusion and broth microdilution methods. Genome-wide comparative analyses were performed to identify the genetic factors driving virulence and antimicrobial drug resistance (e.g., virulence factors, antimicrobial resistance genes (ARGs) and prophage-related sequences). **Results**: Most of the *F. canicola* isolates lacked virulence-associated genes. *F. canicola* is likely resistant to clindamycin, lincomycin and neomycin, but susceptible to penicillin, erythromycin and enrofloxacin. Antimicrobial resistance genes were not found in the *F. canicola* genomes, but prophage-related sequences were identified, suggesting its potential in the transfer of genes associated with drug resistance between bacteria in the oral microbiome. **Conclusions**: *F. canicola* is presumably a commensal organism with low virulence potential, as evidenced by the absence of virulence-associated genes. As *F. canicola* can colonize a wide range of hosts, including humans, further investigation with a greater number of isolates is needed to better understand the role of *F. canicola* in disease development and the spread of drug resistance.

## 1. Introduction

The mammalian oral cavity is colonized by diverse microbial species that co-evolve with the host and adapt to highly dynamic environmental changes. The genetic determinants of host–microbe relations and microbial–microbial interactions that drive the composition of complex polymicrobial communities might play a role in the development of species-specific microbiota [1]. Research on the microbiome of companion animals (mainly dogs and cats) is of great interest, as close daily contact with them opens a way for the transmission of zoonotic diseases. Moreover, when the oral microbial ecosystem is perturbed, the proliferation of potentially pathogenic bacteria leads to oral diseases such as gingivitis, periodontitis and dental caries [2]. Metagenomic analyses of canine saliva reveal several bacterial genera, including *Porphyromonas*, *Prevotella*, *Pasteurella*, *Neisseria*, *Capnocytophaga*, *Conchiformibius*, *Frederiksenia*, *Cutibacterium*, *Actinomyces*, *Campylobacter*, *Desulfomicrobium*, *Bacteroides*, *Fusobacterium*, *Mycoplasmopsis*, *Treponema*, *Streptococcus*, *Bergeyella* and *Histophilus* [3,4], out of which some species are associated with the development of oral or wound infections [5]. Over past decades, the popularity of keeping dogs has been steadily increasing [6]; therefore, accurate species-level identification and correct classification of the members of canine oral microbiota are essential to adequate therapy and disease prevention.

From the majority of dog-bite infections, *Pasteurella* species can be isolated as causative agents [3,7]. *P. multocida*-associated diseases are the most common in humans, although other species of the genus have also been isolated from human infections (*Pasteurella dagmatis*, *Pasteurella canis* and *Pasteurella stomatis*) [8]. *Frederiksenia canicola* is the single representative of the genus *Frederiksenia* (*Pasteurellaceae* family), described in 2014 as a new species previously known as Bisgaard taxon 16 [9]. *F. canicola* might be misidentified as *P. canis*, *P. stomatis* and *P. dagmatis* as it shares phenotypic characteristics, genome size and cellular fatty acid composition with the genus *Pasteurella*. Based on MALDI-TOF mass spectrometry, chemotaxonomy, amino acid signature-specific PCR and phylogenetic analyses of 16S rRNA, *rpoB* (encoding beta subunit of the DNA-dependent polymerase), *infB* (encoding translation initiation factor 2) and *recN* (encoding DNA repair protein), Korczak et al. identified *F. canicola* in dogs, dog-bite wounds, a cat, a lion, a hedgehog and a mongoose [10]. Since then, *F. canicola* has been isolated from Tasmanian devils, long-nosed potoroos, spotted-tailed quolls, eastern quolls and as a component of dog oral microbiota [2,3,11,12,13]. It was also detected in the upper respiratory tract microbiota of humans with and without a severe acute respiratory syndrome coronavirus-2 infection [14]. Of note, despite the high prevalence of *F. canicola* in mammals, especially dogs, clarification of its pathogenic role in the development of infection remains. In addition, the antimicrobial susceptibility profile of this species is largely unknown.

Canine saliva may be a source of antimicrobial resistance genes (ARGs) as well, posing an emerging major concern. ARG-carrying bacteria might enter the human body through biting, scratching, or licking by animals and via contact with nasopharyngeal secretions. Subsequently, ARGs might be exchanged with the host microbiota by horizontal gene transfer. As non-pathogenic bacteria may also harbour acquired antibiotic resistance genes [15], it is essential to assess the genetic characteristics of *F. canicola* along with its potential threats to animal and human health. In this study, we aimed to explore the pathogenic, as well as zoonotic, potential of *F. canicola*. Therefore, we performed antibacterial susceptibility testing of *F. canicola* isolates originating from different body sites (oral cavity, nasal cavity, pharynx and bronchoalveolar lavage) in dogs and carried out a comprehensive genomic analysis to determine possible factors that might contribute to pathogenesis or antimicrobial resistance.

## 2. Results

During 2023, forty-three *F. canicola* isolates have been identified in the oral cavities of healthy dogs. Colonies showed variable phenotypes on Columbia agar plates (small or medium or large in size, greyish or yellowish, smooth and glistening). Isolates were catalase-positive and oxidase-positive, except for three isolates. As breakpoints and susceptible or resistant categories have not yet been established for *F. canicola*, we predicted their susceptibility according to the inhibition zone diameter and low or high minimal inhibitory concentration (MIC) values relative to those of other members of *Pasteurellaceae*. All isolates seemed to be susceptible to the tested antibiotics with the disk diffusion method, except for amoxicillin–clavulanic acid, clindamycin, lincomycin and neomycin (Table 1). One isolate (FC5) was likely resistant to ampicillin, whereas two isolates (FC6 and FC8) showed reduced susceptibility to erythromycin. One isolate (FC5) showed moderate susceptibility to cephalexin and another (FC8) to sulfamethoxazole–trimethoprim. In general, the susceptibility of isolates by broth microdilution was consistent with disk diffusion. The majority of isolates showed low MIC values for ampicillin (0.25–2 mg/L), erythromycin (<0.125–0.5 mg/L), enrofloxacin (≤0.03–0.25 mg/L), penicillin (≤0.03–0.25 mg/L) and tetracycline (0.25–2 mg/L). FC5 and FC10 had MIC values suggestive of the nonsusceptible category to ampicillin. FC14 had reduced susceptibility to tetracycline (2 mg/L) and enrofloxacin (0.25 mg/L). The lowest MIC values were observed for enrofloxacin with MIC_90_ ≤ 0.03 mg/L. All *F. canicola* isolates had relatively high MIC values for clindamycin (4–32 mg/L) and lincomycin (8–128 mg/L). Neomycin proved to be effective in vitro only against FC3 (0.25 mg/L); otherwise, it demonstrated weak activity against *F. canicola* isolates (4–64< mg/L) (Table 1).

**Figure 1:**
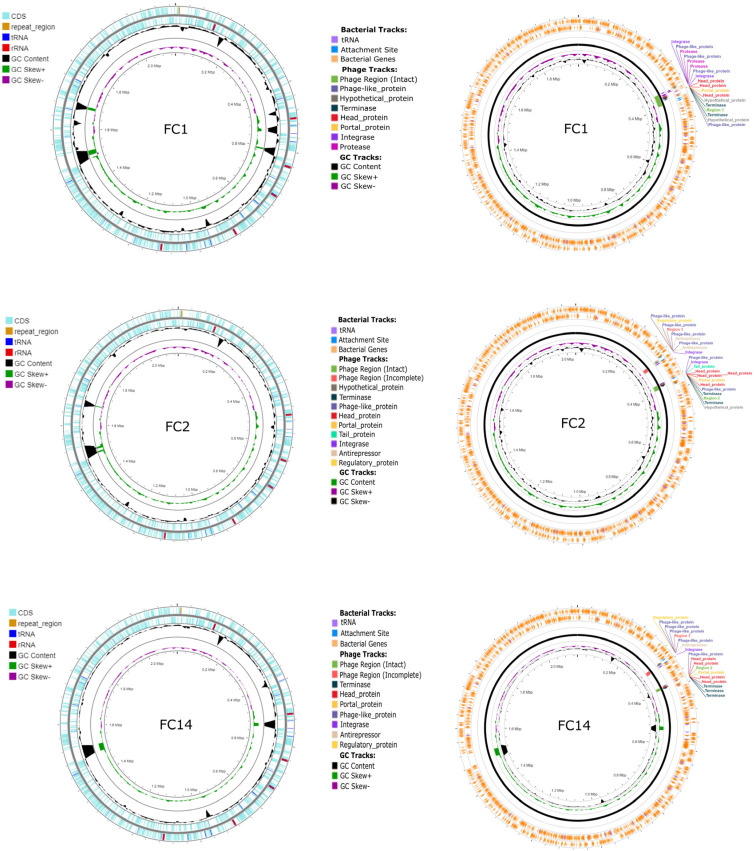
A comparative chromosomal genome visualization of the *F. canicola* isolates FC1, FC2 and FC14, generated by Proksee and PHASTEST. On the left, circular genomes illustrate coding sequences (CDS), repeat regions, tRNAs, rRNAs, GC content and GC skew. On the right, genomes illustrate the location of phage-related genes (terminase, head protein, portal protein, phage-like protein, integrase, antirepressor, regulatory protein, tail protein and protease).

**Figure 2:**
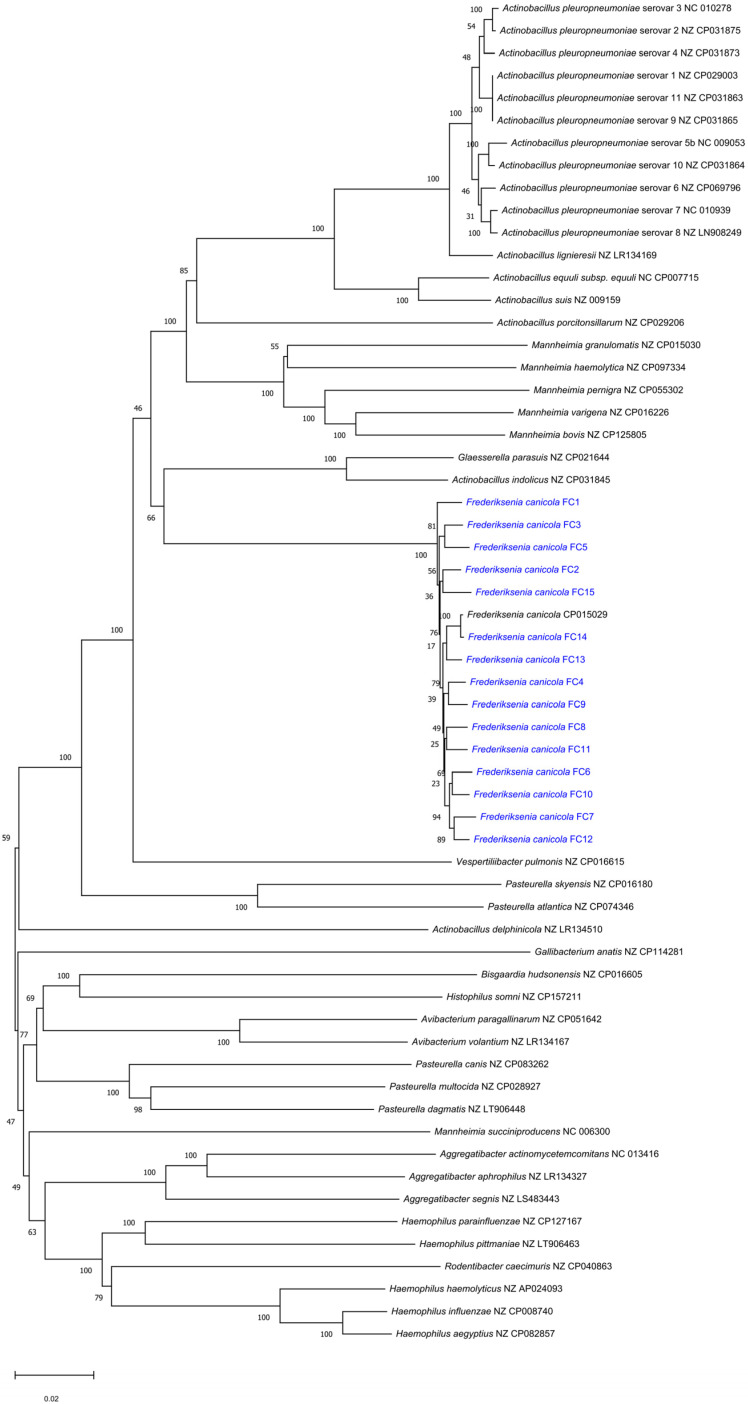
The phylogenetic relationships of bacterial isolates representing the members of the *Pasteurellaceae* family. The tree was generated by the neighbor-joining algorithm with the p-distance method based on concatenated sequences of 16S rRNA, *rpoB*, *infB* and *recN* genes (9812 bp). The numbers along the branches indicate bootstrap values. The *F. canicola* isolates involved in the study are marked in blue.

The sequencing of *F. canicola* genomes resulted in 22,010,483–28,422,845 reads with 1284–1711.9-fold mean coverage of the whole genome. We determined that the average nucleotide identity between the sequenced *F. canicola* genomes and the reference sequence was 92.92%, reflecting the degree of evolutionary distance between the genomes. ARGs were not found in the investigated genomes; however, SNP in 23S rRNA conferring resistance to clindamycin and mutation in 16S rRNA conferring resistance to spectinomycin were detected in all isolates using the Bacterial and Viral Bioinformatics Resource Center (BV-BRC) database. In FC14, SNP in 23S rRNA, associated with resistance to macrolides and streptogramins, was also identified. Based on the Comprehensive Antibiotic Resistance Database (CARD) and Resistance Gene Identifier (RGI), SNP in the elongation factor Tu (EF-Tu), yielding resistance to pulvomycin, was detected in all *F. canicola* sequences. Known virulence factors were not identified by BV-BRC. In FC1, FC3, FC4 and FC5, a heat-shock protein sequence (*htpB*) was identified with VRprofile2. The sequence of undecaprenyl-phosphate galactose phosphotransferase (*wbaP*/*rfbP*), involved in O-antigen biosynthesis, was found only in FC14. The *F. canicola* genomes FC1, FC2 and FC14 contained complete prophage regions with lengths of 40.2 Kb, 16.5 Kb and 9.6 Kb, respectively. FC6, FC8, FC9, FC10, FC11 and FC13 harboured questionable prophages (a length range of 9.6–34.8 Kb). Moreover, an incomplete prophage was found in FC2 (12.6 Kb) and FC14 (12.4 Kb) (Table 2, Figure 1). Phylogenetic analysis of the concatenated sequences, including whole 16S rRNA, *rpoB*, *infB* and *recN* genes, revealed that the *F. canicola* isolates were highly similar in comparison with each other or the reference strain HPA 21. The 9812-bp-long sequences of isolates obtained in our study demonstrated 98.7% pairwise identity with 9359 identical sites (95.4%). The *F. canicola* gene sequences formed a monophyletic group and bore the closest relation to *Glaesserella parasuis* and *Actinobacillus indolicus* (Figure 2).

## 3. Discussion

Most antibiotics are used in both human and veterinary medicine; thus, the One Health approach is inevitable in adequate disease management and control of the spread of antimicrobial resistance. Since animals and their owners can exchange microbiota and even non-pathogenic bacterial strains may acquire and maintain ARGs [16], the identification and examination of animal microbial populations that can endanger animal and human health is of great importance. In 2014, a new species was described within the *Pasteurellaceae* family, named *Frederiksenia canicola* [10]. The widespread use of next-generation sequencing technologies over the last decade has allowed for the identification of this species in various hosts, with an outstanding prevalence in the oral cavities of dogs. Lisjak et al. reported that the most abundant bacteria in the oral microbiota of healthy dogs were the *Porphyromonas* species and *Conchiformibius steedae*, followed by *Neisseria weaveri* and *F. canicola*. Interestingly, *Frederiksenia* was present in high frequency in dogs with oral tumours, along with the *Bergeyella* and *Pasteurella* species [4]. In another study, the most prevalent oral bacteria at the genus level were *Porphyromonas*, *Bergeyella*, *Capnocytophaga*, *Pasteurella*, *Tannerella*, *Neisseria*, *Desulfomicrobium*, *Frederiksenia*, *Treponema*, *Conchiformibius*, *Fusobacterium*, *Campylobacter* and *Pseudomonas* [17]. In addition, 16S rRNA coding gene sequences of *F. canicola* have been detected in the upper respiratory tract microbiota of asymptomatic individuals, as well as patients with confirmed COVID-19, suggesting that *F. canicola* can colonize a wide range of hosts, including humans [14].

Only a few data regarding the antimicrobial susceptibility of *F. canicola* are available in the scientific literature. Gutman et al. investigated the susceptibility of the members of the *Pasteurellaceae* family, including *F. canicola*, originated in the oral cavities of Tasmanian devils. All *F. canicola* isolates demonstrated MIC values > 32 mg/L for amikacin, whereas penicillin proved to be the most efficient drug (MIC range: ≤0.06–0.25 mg/L) [18]. In our study, we investigated the susceptibility of *F. canicola* isolates with the disk diffusion and broth microdilution methods and obtained similar results to Gutman et al.; one of the most effective drugs was penicillin G (MIC_90_ = 0.125 mg/L). However, the lowest MIC values were observed for enrofloxacin (MIC_90_ =≤ 0.03). Gutman et al. examined the activity of enrofloxacin and also found low MIC values [18]. Nevertheless, the lowest tested concentration was 0.25 mg/L, in contrast to our study (0.03 mg/L). Instead of amikacin, we chose neomycin, from the aminoglycoside drug class, for our susceptibility testing and found that inhibition of bacterial growth could be seen at high concentrations (MIC_90_ = 16 mg/L). Our observations, and the overall high prevalence of strains from the *Pasteurellaceae* family with high MIC values for amikacin and gentamicin, further support that these strains possess resistance mechanisms to aminoglycosides [18,19]. The highest MIC values were seen for clindamycin (MIC_90_ = 16 mg/L) and lincomycin (MIC_90_ = 32 mg/L), which belong to the lincosamide drug class; hence, resistance to these agents is presumed. Although resistance cannot be declared in the absence of breakpoints, the SNPs found in genes encoding 23S rRNA and 16S rRNA confirmed these hypotheses. Spectinomycin is an aminocyclitol antibiotic that has a similar chemical structure and mechanism of action to aminoglycosides; thus, a mutation in 16S rRNA might explain the reduced activity of aminoglycosides against *F. canicola*. Overall, *F. canicola* isolates are likely susceptible to ampicillin, penicillin, tetracycline, erythromycin, enrofloxacin, cefalexin, cefovecin and sulfamethoxazole–trimethoprim.

Several bacterial genomes harbour regions that include prophage-associated genes acting as hotspots for horizontal gene transfer and thereby contribute to host adaptation, virulence, stress tolerance and antimicrobial resistance [20,21]. A lambda-like phage carrying the toxin-encoding gene *toxA* and other prophage-associated genes were detected in the genome of toxigenic *P. multocida* serogroup D strains, suggesting that these genes might influence the short-term evolution of *P. multocida* [22]. We identified complete prophage regions in three out of fifteen of the *F. canicola* isolates and additional incomplete prophages in two isolates. Only questionable prophage regions were detected in the other isolates. As intact and incomplete prophage regions consisted of genes specific to phage-inducible chromosomal islands (e.g., integrase and transcriptional regulator) found in pathogenic bacteria, *F. canicola* isolates may have the potential to play some role in the transfer of resistance genes due to carrying prophages, although we could not detect any genes contributing to drug resistance.

Virulence factors associated with pathogenicity were identified in bacterial species by genomic analyses. Understanding the role of these factors in pathogenic mechanisms facilitates the prevention and control of infection. Species within the *Pasteurellaceae* family share several genes associated with virulence that often participate in the biosynthesis of lipopolysaccharide and adhesins, iron regulation, or help bacteria to evade the host’s innate immune system. Gong et al. investigated the genome of 764 *G. parasuis* isolates and identified 24 potential virulence factors, all of which linked to lipooligosaccharides. Thirteen of these genes (*galE*, *galU*, *gmhA*, *isgF*, *kdsA*, *lgtF*, *lpxA*, *lpxC*, *lpxD*, *neuA*, *rfaD*, *rfaE* and *yhxB*/*manB*) were present in all the *G. parasuis* isolates [23]. Some genes, required for the biosynthesis of lipid A (*lpxA*, *lpxC*, *lpxD* and *kdsA*) and inner core oligosaccharide (*gmhA*), were detected in *P. multocida* strains, as well [21]. Besides these conserved virulence factors in the *G. parasuis* genome, there were specific genes whose presence correlated with different serovars. Previously, the presence of *lsgB*-encoding sialyltransferase was counted specifically in isolates that cause systemic infections; however, Gong et al. reported that nasal isolates also carried the *lsgB* gene. In addition, *lsgB* was mainly detected in highly virulent *G. parasuis* strains belonging to serovars 7–10 and 13 [23]. We only found a heat-shock protein sequence (*htpB*) in four of the *F. canicola* isolates and an undecaprenyl-phosphate galactose phosphotransferase (*wbaP*/*rfbP*) in one isolate. As *F. canicola* shows the closest phylogenetic relationship with *G. parasuis* and *A. indolicus* and we did not identify any of the above-mentioned virulence genes or other virulence-associated factors, such as adhesins, toxins, iron-regulated, iron acquisition proteins, nor enzymes involved in the sialic acid metabolism, we conclude *F. canicola* strains are likely non-virulent.

## 4. Materials and Methods

### 4.1. Sample Collection and Identification

During 2023, oral swab samples were collected from healthy dogs representative of different sexes, breeds and ages (Table 3). The swab samples were collected by the veterinarian working at the practice, with the consent of the animals’ owners, and in full compliance with the ethical guidelines outlined in the code of ethics of the HUN-REN Veterinary Medical Research Institute. The samples were cultured on Columbia agar plates supplemented with 5% sheep’s blood and incubated at 37 °C for 24 h under aerobic conditions. *Pasteurella*-like colonies were further investigated by biochemical testing and molecular methods. Five isolates from our *F. canicola* strain collection were also involved in the study. Species-specific PCR testing was carried out with the primers recN_Fred-1 CCACGCTCTATCAAACTATTCG and recN_first-R CCRCTAATYCCMACATCNACYTCATC amplifying a 747 bp fragment of a *recN* gene. Briefly, the genomic DNA template used in the PCR tests was obtained from bacterial colonies by heat inactivation. The 25 µL reaction mixture contained 1 µL bacterial DNA, 2.5 µL 10× DreamTaq buffer, 0.3 µL MgCl_2_ (25 mM), 0.5 µL dNTP (10 mM), 0.3 µL forward and reverse primers (10 µM each), 0.2 µL DreamTaq DNA polymerase (5 U/µL; Thermo Fisher Scientific, Waltham, MA, USA) and 19.9 µL distilled water. The PCR condition was set up with an initial denaturation step at 94 °C for 3 min, followed by 30 cycles of 94 °C for 15 s, annealing at 50 °C for 20 s, extension at 72 °C for 30 s and a final extension step at 72 °C for 2 min. Signature sequence-specific amplicons for *F. canicola* were verified on a 1.5% agarose gel [10].

### 4.2. Antibiotic Susceptibility Testing

The in vitro susceptibility of *F. canicola* isolates was evaluated by disk diffusion and broth microdilution methods according to Clinical and Laboratory Standards Institute (CLSI) guidelines [24,25]. The antibiotics involved in the study were chosen based on the following criteria: the most antibiotic classes are represented, including agents used in small-animal practices in Hungary, and those antibiotics previously used in the susceptibility testing of *F. canicola* in other publications. The initial concentration of bacterial cell suspension in sterile saline was adjusted to an optical density of 0.5 McFarland for both susceptibility methods. Disk diffusion was performed on Mueller–Hinton agar plates supplemented with 5% sheep’s blood. Commercially prepared paper disks containing a fixed concentration of antibiotics were applied to the inoculated agar. The following antibiotics were tested: penicillin G (10 U), amoxicillin + clavulanic acid (20/10 µg), ampicillin (10 µg), neomycin (30 U), lincomycin (2 µg), cephalexin (30 µg), cefovecin (30 µg), clindamycin (2 µg), enrofloxacin (5 µg), erythromycin (15 µg), sulfamethoxazole–trimethoprim (25 µg) and tetracycline (30 µg). The results were analysed 24 h after inoculation. *Escherichia coli* ATCC 25922 and *Staphylococcus aureus* ATCC 25923 strains were used as quality controls. In the broth microdilution method, the activity of penicillin (0.03–16 mg/L), ampicillin (0.125–64 mg/L), clindamycin (0.25–128 mg/L), erythromycin (0.125–64 mg/L), lincomycin (0.25–128 mg/L), neomycin (0.125–64 mg/L), cephalexin (0.03–16 mg/L), enrofloxacin (0.03–16 mg/L) and tetracycline (0.06–32 mg/L) against *F. canicola* was tested. Twofold dilutions of the drug solutions in Brain Heart Infusion (BHI) broth were dispensed into 96-well plates. *E. coli* ATCC 25922 and *S. aureus* ATCC 29213 were used as quality control strains to check the reproducibility of the procedure. The plates were incubated at 37 °C for 24 h. The growth of each isolate of various drug concentrations was read at 450 nm using a microplate reader and the results were evaluated in comparison to the growth of isolates in a drug-free medium. The MIC endpoint was defined as the lowest concentration that completely inhibited bacterial growth. The concentration of antibiotics that inhibited the growth of 90% of the tested isolates was considered the MIC_90_ value.

### 4.3. Whole-Genome Sequencing and Bioinformatical Analyses

Genomic DNA was extracted with a Quick-DNA Fungal/Bacterial Miniprep Kit (Zymo Research, Irvine, CA, USA), in accordance with the manufacturer’s instructions, after a 24-h incubation of the *F. canicola* isolates in BHI broth at 37 °C. Ten isolates from the oral cavities of dogs collected in 2023 and five isolates from our culture collection obtained in 2018–2019 were chosen for sequencing purposes. Sequencing of the whole genome of the isolates was performed by SeqOmics Biotechnology Ltd. (Mórahalom, Hungary) on an Illumina platform. Paired-end reads were quality-checked and trimmed using the BBDuk algorithm in Geneious Prime (version 2023.2.1) [26]. Genome assembly was carried out by mapping the trimmed reads to the reference *(F. canicola* strain HPA 21, GenBank accession number: CP015029) using Geneious Prime software. The characteristics of bacterial genomes (e.g., genes, GC content) were visualized by Proksee (https://proksee.ca/ (accessed on 15 July 2024)). ARG sequences or SNPs conferring antibiotic resistance were assessed in paired reads and their genomes assembled with the BV-BRC (https://www.bv-brc.org/ (accessed on 1 July 2024)) and the CARD RGI (https://card.mcmaster.ca/analyze/rgi (version RGI 6.0.3, CARD 3.3.0, accessed on 10 July 2024)), respectively. Only those genes or SNPs that met the STRICT threshold criteria established by the CARD database and a query and template sequence pairwise identity of ≥90% were taken into account. Potential virulence factors were determined with the BV-BRC and VRprofile2 (https://tool2-mml.sjtu.edu.cn/VRprofile/VRprofile.php (accessed on 1 July 2024)). Prophage content in the *F. canicola* genomes was estimated with the PHASTEST web server (https://phastest.ca/ (accessed on 17 July 2024)). Prophage sequences were categorized as intact (score of >90), questionable (score of 70–90) and incomplete (score of <70). The phylogenetic relationship between the *F. canicola* isolates and other members of the *Pasteurellaceae* family, based on concatenated sequences of 16S rRNA, *rpoB*, *infB* and *recN*, were reconstructed with the neighbor-joining algorithm and p-distance model, supported by bootstrapping, with 1000 replications, as implemented in MEGA 11 [27]. One isolate with publicly available complete genome sequences from each species belonging to the *Pasteurellaceae* family was chosen for phylogenetic analysis.

## 5. Conclusions

Although we examined a low number of isolates, to the best of our knowledge, this study is the first comparative genome analysis of *F. canicola* isolates that provides deeper insight into the genetic factors driving virulence or antimicrobial drug resistance. Virulence-associated genes that might contribute to pathogenesis were not found in any of the investigated *F. canicola* genomes; therefore, *F. canicola* is presumed a commensal bacterial organism in the oral cavities of dogs rather than a pathogen. *F. canicola* is likely resistant to clindamycin, lincomycin and neomycin, however, penicillin, erythromycin and enrofloxacin showed excellent antibacterial activity against the isolates. ARGs were not detected in the *F. canicola* genomes; nevertheless, prophage-related sequences were identified, and so the possibility of resistance gene transfer between *F. canicola* and other oral bacterial strains cannot be ruled out. Despite the high genome-wide pairwise nucleotide identity of the *F. canicola* isolates, further investigation, including a greater number of isolates, as well as clinical samples, is needed to elucidate its role in disease development and the spread of drug resistance.

## Figures and Tables

**Table 1:** Antibiotic susceptibility of *F. canicola* isolates determined by disk diffusion and broth microdilution methods. Numbers under disk diffusion represent diameter (mm) and numbers under BMD represent mg/L. Green, yellow and red colours indicate susceptible, intermediate and resistant categories, respectively, according to CLSI guidelines, based on *Pasteurella* species.

Isolates	Ampicillin		Amoxicillin–Clavulanic Acid		Penicillin G		Neomycin		Tetracycline		Erythromycin		Enrofloxacin		Clindamycin		Lincomycin		Cefalexin		Cefovecin		SXT	
	Disk	BMD	Disk	BMD	Disk	BMD	Disk	BMD	Disk	BMD	Disk	BMD	Disk	BMD	Disk	BMD	Disk	BMD	Disk	BMD	Disk	BMD	Disk	BMD
FC1	34.3	0.25	26	ND	32	0.06	13.7	16	30	0.25	30.3	0.5	36.3	0.06	19.3	16	0	8	30.7	2	37.3	ND	32	ND
FC2	ND	0.5	ND	ND	ND	0.125	ND	8	ND	0.5	ND	0.25	ND	≤0.03	ND	16	ND	16	ND	4	ND	ND	ND	ND
FC3	35.3	≤0.125	34.3	ND	41.7	≤0.03	22	0.25	31.7	0.5	29	0.25	47.3	≤0.03	17.7	8	0	16	26.7	2	31.3	ND	28.3	ND
FC4	ND	0.5	ND	ND	ND	0.25	ND	8	ND	1	ND	0.25	ND	≤0.03	ND	32	ND	32	ND	4	ND	ND	ND	ND
FC5	26.3	2 *	23	ND	29.7	0.25	20	4	30.7	0.5	30	0.25	27.7	≤0.03	0	16	0	16	20	16	38	ND	38.7	ND
FC6	28.5	0.5	25.7	ND	29.7	0.125	16.2	8	26.5	0.5	24.8	0.25	37.7	≤0.03	13	16	7.3	16	26.3	8	32.7	ND	31.3	ND
FC7	31.3	0.5	23	ND	31.3	0.06	12.8	8	29.2	0.5	28.3	0.25	31	≤0.03	13.2	16	0	16	25.3	2	29.3	ND	24.3	ND
FC8	30.3	≤0.125	23	ND	29.7	0.06	15.7	16	27.3	1	26.8	0.25	37.8	≤0.03	11.5	16	0	128	26	2	30.3	ND	23.7	ND
FC9	30.3	≤0.125	30	ND	37.3	0.06	15.3	16	26.8	0.25	28	<0.125	39.5	≤0.03	11	16	0	8	34	2	36.7	ND	39.3	ND
FC10	29.7	2 *	25.7	ND	31	0.06	14.3	>64	33.3	0.5	27	0.25	37.7	≤0.03	15.7	16	0	16	27.7	2	31.7	ND	31	ND
FC11	35.7	0.25	26.3	ND	35.7	0.125	16.3	8	31.3	0.5	28.3	<0.125	40.7	≤0.03	20.7	4	0	8	30.7	2	40	ND	35.7	ND
FC12	30.7	≤0.125	32.3	ND	34.8	0.125	16.8	16	32	0.5	29.2	0.5	40.5	≤0.03	16	16	10.8	16	28.7	4	33	ND	26.7	ND
FC13	ND	0.25	ND	ND	ND	0.125	ND	16	ND	1	ND	0.25	ND	≤0.03	ND	32	ND	32	ND	2	ND	ND	ND	ND
FC14	ND	≤0.125	ND	ND	ND	0.125	ND	64	ND	2 *	ND	<0.125	ND	0.25 *	ND	16	ND	16	ND	4	ND	ND	ND	ND
FC15	ND	0.25	ND	ND	ND	0.06	ND	16	ND	1	ND	<0.125	ND	≤0.03	ND	16	ND	16	ND	2	ND	ND	ND	ND

BMD: broth microdilution, ND: not determined, SXT: sulfamethoxazole–trimethoprim, *: suggestive of a “nonsusceptible” category.

**Table 2:** Characteristics of prophage regions identified in *F. canicola* genomes.

Isolate	Completeness of Prophage	Region Position	Region Length (Kb)	Number of Total Prophage Protein	GC Content (%)
FC1	Intact	344,931–385,214	40.2	26	43.98
FC2	Intact	365,047–381,552	16.5	12	40.86
	Incomplete	292,040–304,657	12.6	15	44.09
FC6	Questionable	381,721–399,394	17.6	13	36.32
	Questionable	1,491,354–1,526,192	34.8	31	23.95
FC8	Questionable	381,652–391,296	9.6	9	35.27
FC9	Questionable	381,734–394,626	12.8	13	30.72
FC10	Questionable	381,689–399,427	17.7	9	31.38
FC11	Questionable	381,741–399,462	17.7	12	31.32
FC13	Questionable	381,662–393,451	11.7	12	32.78
FC14	Intact	381,739–391,387	9.6	11	35.41
	Incomplete	306,552–318,970	12.4	17	42.64

**Table 3:** The origin of the sequenced *F. canicola* isolates and the distribution of the age, sex and breed of the studied population of healthy dogs.

Isolate Identifier	Date of Isolation	Sampling Site	Breed	Age	Sex	Location
FC1	2023	Oral cavity	French Bulldog	4 years	Female	Kiskunfélegyháza
FC2	2019	Pharynx	NA	NA	NA	NA
FC3	2023	Oral cavity	Havanese	11 years	Female	Jásszentlászló
FC4	2018	BAL	NA	NA	NA	NA
FC5	2023	Oral cavity	Hungarian Greyhound	14 months	Male	Budapest
FC6	2023	Oral cavity	Weimaraner	3 years	Female	Miskolc
FC7	2023	Oral cavity	Large Munsterlander	4 years	Male	Mátraderecske
FC8	2023	Oral cavity	Dachshund	1.5 years	Male	Kecskemét
FC9	2023	Oral cavity	German Pointer	8 years	Female	Budapest
FC10	2023	Oral cavity	Cross-breed	2 years	Female	Kiskunfélegyháza
FC11	2023	Oral cavity	Border Collie	9 years	Male	Gyál
FC12	2023	Oral cavity	Sheltie	3 months	Male	Kaposvár
FC13	2018	Pharynx	NA	NA	NA	NA
FC14	2018	Nasal cavity	NA	NA	NA	NA
FC15	2018	Pharynx	NA	NA	NA	NA

NA: not available.

## Data Availability

Raw reads of the sequenced *F. canicola* isolates have been deposited to the NCBI Sequence Read Archive under BioProject ID PRJNA1161829.
