# Correction: Domán et al. Comparative Genome Analysis of Canine *Frederiksenia canicola* Isolates. *Antibiotics* 2024, *13*, 1235

**DOI:** 10.3390/antibiotics14040369

**Published:** 2025-04-02

**Authors:** Marianna Domán, Krisztina Pintér, Boglárka Dóra Pollák, Ágnes Pintér, Enikő Wehmann, Miklós Tenk, Tibor Magyar

**Affiliations:** 1 HUN-REN Veterinary Medical Research Institute, 1143 Budapest, Hungary; 2 Department of Microbiology and Infectious Diseases, University of Veterinary Medicine, 1143 Budapest, Hungary; 3 National Laboratory of Infectious Animal Diseases, Antimicrobial Resistance, Veterinary Public Health and Food Chain Safety, University of Veterinary Medicine, 1078 Budapest, Hungary

## Additional Affiliation(s)

In the published publication [1], there was an error regarding the affiliation(s) for Enikő Wehmann, Miklós Tenk, and Tibor Magyar. In addition to affiliation(s) 1, 2, the updated affiliation(s) should include the following: 3 National Laboratory of Infectious Animal Diseases, Antimicrobial Resistance, Veterinary Public Health and Food Chain Safety, University of Veterinary Medicine, 1078 Budapest, Hungary. The authors state that the scientific conclusions are unaffected. This correction was approved by the Academic Editor. The original publication has also been updated.
